# Long noncoding RNA AB073614 promotes the malignance of glioma by activating Wnt/β-catenin signaling through downregulating SOX7

**DOI:** 10.18632/oncotarget.19305

**Published:** 2017-07-17

**Authors:** Yuqian Li, Gang Zhu, Wen Zeng, Jiancai Wang, Zhihong Li, Bao Wang, Bo Tian, Dan Lu, Xingye Zhang, Guodong Gao, Lihong Li

**Affiliations:** ^1^ Department of Neurosurgery, Tangdu Hospital, The Fourth Military Medical University, Xi’an, Shaanxi, 710038, China

**Keywords:** AB073614, glioma, long noncoding RNA, SOX7, Wnt/β-catenin

## Abstract

Long noncoding RNA (lncRNA) AB073614 has recently shown to be aberrantly increased and identified as a poor prognostic biomarker in human glioma. However, the potential mechanisms remain unknown. This study demonstrated that AB073614 expression was significantly upregulated in both glioma tissues and cell lines, and glioma patients with high AB073614 expression had lower overall survival rates. Importantly, silencing AB073614 expression remarkably inhibited U251 cell proliferation, migration, and invasion *in vitro*, as well as suppressed tumor formation *in vivo*. The level of AB073614 was found to correlate inversely with sex-determining region Y-box 7 (SOX7) expression but correlate positively with Wnt/β-catenin signaling activity. Of note, the data showed that the inhibition of SOX7 could reverse the tumor-suppressive effect of the silencing AB073614 on glioma *in vitro* and *in vivo*. Furthermore, the results indicated that AB073614 induced Wnt/β-catenin signaling activity by repressing SOX7 expression. In conclusion, the findings demonstrated that AB073614 promoted the progression of glioma by targeting SOX7 to activate the Wnt/β-catenin signaling pathway, suggesting that the inhibition of AB073614 might be a potential target for therapeutic intervention in glioma patients.

## INTRODUCTION

Glioma is the most common and aggressive form of adult primary brain tumors, accounting for 80% of malignant brain tumors [1, 2]. According to the degree of malignancy, glioma is generally categorized into four histopathological grades [World Health Organization (WHO) grade I–IV] [3]. Despite recent advances in the treatment of glioma, including surgical resection followed by chemotherapy and radiotherapy, the prognosis, especially for patients with glioblastoma (GBM, WHO grade IV), remains extremely dismal with a mean life expectancy of only 9–12 months and the 5-year survival rate less than 10% [4]. A major barrier to effective treatment of glioma is its high proliferation, progressive diffusion, and invasion nature, yet the underlying mechanisms governing glioma remain far from understanding. Therefore, it is urgently needed to investigate the novel molecular mechanisms and establish more effective and useful therapeutic methods.

Long noncoding RNAs (lncRNAs) are a class of transcripts longer than 200 nucleotides in length with no protein-coding ability [5]. Dysregulation of lncRNAs has been observed in several human diseases, especially in malignancies. Accumulating evidences indicate that aberrantly expressed lncRNAs contribute to tumorigenesis and cancer progression [6–8]. Recently, lncRNA AB073614 is reported to act as an oncogene in ovarian cancer development [9]. Notably, increased lncRNA AB073614 expression is found in glioma and identified as a poor prognostic biomarker [10]. However, the potential mechanisms underlying the tumorigenic role of AB073614 in human glioma remain unknown.

The Wnt/β-catenin signaling pathway is a highly conserved molecular mechanism that regulates a variety of biological processes, such as cell proliferation, differentiation, and morphogenesis in both normal development and disease progression [11–13]. Moreover, it was reported to contribute to the formation of glioma [14, 15]. Sex-determining region Y-box 7 (SOX7), one of the SOX transcription factors family, has been recently recognized as a tumor suppressor implicated in human cancers. The SOX7 gene encodes a variety of transcription factors involved in regulating embryonic development and determining the cell fate [16, 17]. Increasing lines of evidence have shown that SOX7 may play pivotal roles in multiple cancers via regulating the activity of Wnt/β-catenin signaling pathway [18–20].

The present study aimed to investigate the role and possible molecular mechanism of AB073614 in human glioma evolution. The results showed that the expression of AB073614 was significantly increased in both glioma tissues and cell lines; it was inversely correlated with SOX7 but positively correlated with Wnt/β-catenin signaling activity. Importantly, silencing AB073614 could not only remarkably suppress Wnt/β-catenin transcription and its targeted genes, including Cyclin D1 and c-Myc, but also inhibit cell proliferation, migration, and invasion *in vitro*. However, the inhibition of SOX7 could reverse the tumor-suppressive effect of silencing AB073614. These results suggested that AB073614 promoted the progression of glioma by activating Wnt/β-catenin signaling through downregulating Sox7, and the inhibition of AB073614 might be a potential therapeutic approach for treating glioma.

## RESULTS

### High expression of AB073614 was correlated with poor outcome of glioma patients

**Figure 1:**
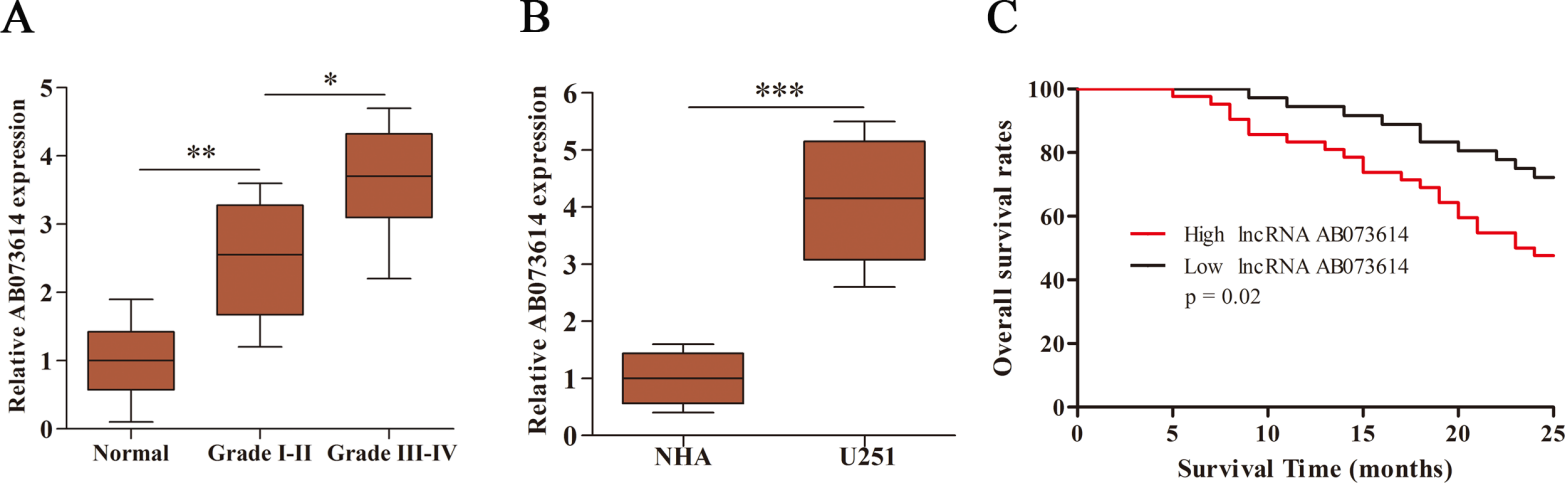
LncRNA AB073614 was highly expressed in glioma and its relationship with overall survival of glioma patients (**A**) Relative expression of AB073614 was measured using qRT-PCR in glioma and adjacent normal tissues (*n* = 89). AB073614 expression level was correlated positively with the WHO grade. (**B**) Relative expression of AB073614 was measured using qRT-PCR in NHA and U251 cells. (**C**) Kaplan-Meier analyses of the relationship between AB073614 expression level and overall survival rates of glioma patients (*P < 0.05; **P < 0.01; ***P < 0.001).

Here, we first investigated the expression level of AB073614 in human glioma tissues and cells. The expression level of AB073614 was detected in 89 paired human glioma and adjacent normal tissues, as well as in glioma cell line (U251) and normal human astrocytes (NHA), using quantitative real-time polymerase chain reaction (qRT-PCR). AB073614 expression was found to be significantly upregulated in glioma tissues compared with adjacent normal tissues, with a higher level in high-grade glioma (Figure 1A). In parallel, the expression level of AB073614 was also greatly upregulated in U251 cells compared with NHA (Figure 1B). Further, we evaluated whether the expression level of AB073614 was correlated with the overall survival rates of glioma patients. The Kaplan–Meier method and log-rank test revealed that high AB073614 expression was inversely correlated with the overall survival rates of glioma patients (Figure 1C).

### AB073614 regulated the activity of Wnt/β-catenin signaling pathway in glioma cells

**Figure 2:**
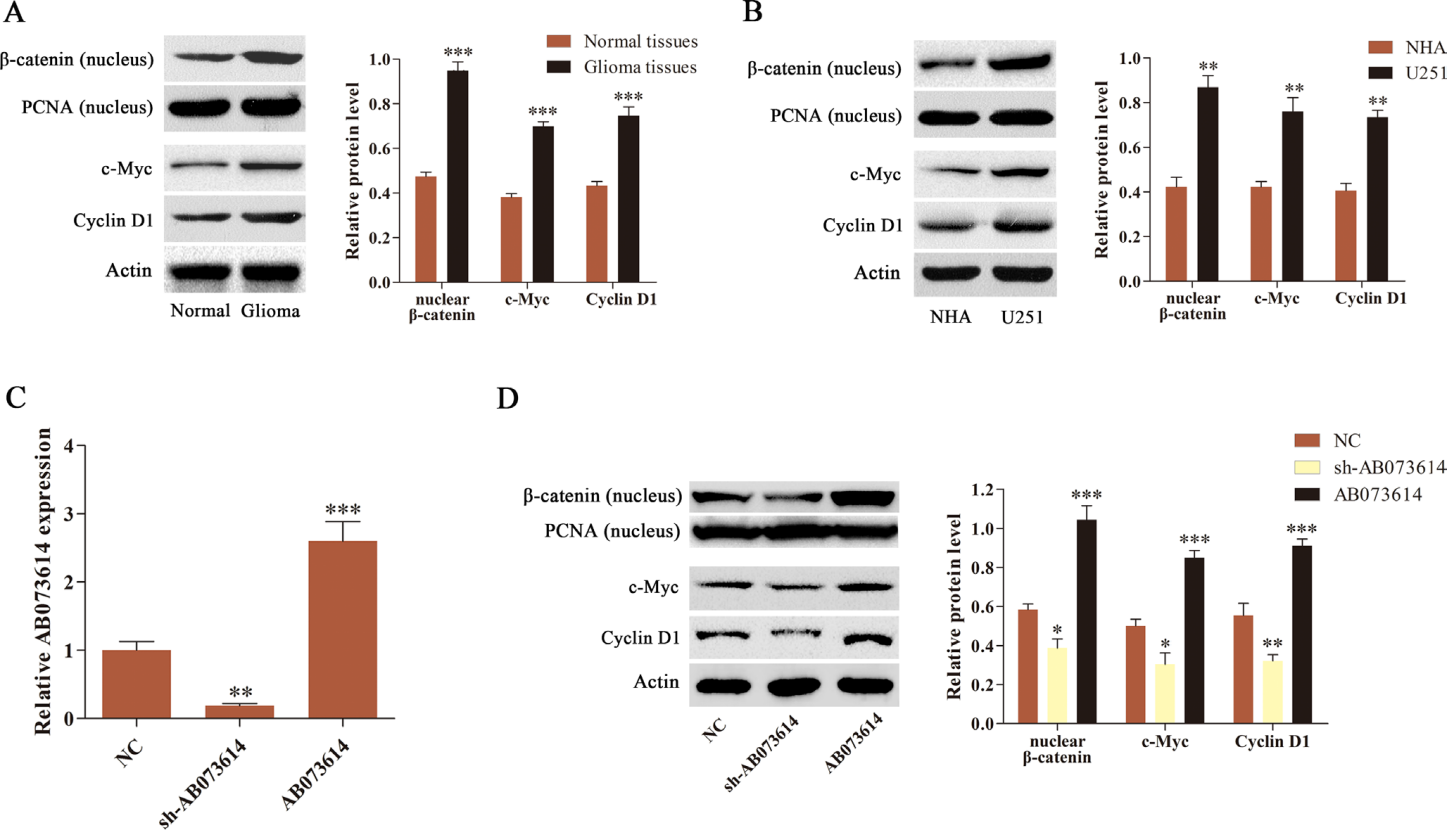
AB073614 was able to positively regulate the Wnt/β-catenin signaling activity (**A**) Western blot analysis of the level of nuclear β-catenin and its target gene (Cyclin D1 and c-Myc) expression in glioma and adjacent normal tissues (*n* = 89). (**B**) Western blot analysis of the level of nuclear β-catenin and its target gene (Cyclin D1 and c-Myc) expression in NHA and U251 cells. (**C**) The AB073614 expression level in U251 cells transduced with NC, sh-AB073614, or AB073614 was measured using qRT-PCR. (**D**) Western blot analysis showed a remarkable decrease in the level of nuclear β-catenin and its target gene (Cyclin D1, c-Myc) expression in U251 cells transduced with sh-AB073614, but a significantly increased expression of the three proteins in U251 cells transduced with AB073614 compared with the NC group (*P < 0.05; **P < 0.01; ***P < 0.001).

The study then explored whether the Wnt/β-catenin signaling pathway was activated in human glioma. We isolated cytoplasm and nucleus, and measured the expression of β-catenin in nucleus, as well as two Wnt/β-catenin signaling targets Cyclin D1 and c-Myc in the whole cell by western blot assay in this study. The results showed that the level of nuclear β-catenin and its target gene (Cyclin D1 and c-Myc) expression was markedly increased in glioma tissues and cell lines compared with normal tissues or NHA, respectively (Figure 2A and 2B). U251 cells were then transduced with shRNA targeting AB073614 (sh-AB073614), one negative control (NC), and an AB073614 overexpression (AB073614) lentiviral vector to investigate the association of AB073614 with Wnt/β-catenin signaling activity in glioma. The results from the qRT-PCR analysis showed that AB073614 expression was downregulated in the sh-AB073614 group and upregulated in the AB073614 group compared with the NC group, indicating a successful modulation of AB073614 in U251 cells (Figure 2C). Then, western blot analysis was carried out to evaluate the level of nuclear β-catenin, and its target gene (Cyclin D1 and c-Myc) expression. The inhibition of AB073614 caused remarkably decreased expression of the three proteins, however a signifcant increase was found in the AB073614 group (Figure 2D). The data indicated that the activity of Wnt/β-catenin signaling pathway was positively correlated with the AB073614 expression level, and AB073614 might be a positive regulator of Wnt/β-catenin signaling activity in glioma cell lines.

### Silencing AB073614 suppressed cell proliferation, migration, and invasion of glioma cells

**Figure 3:**
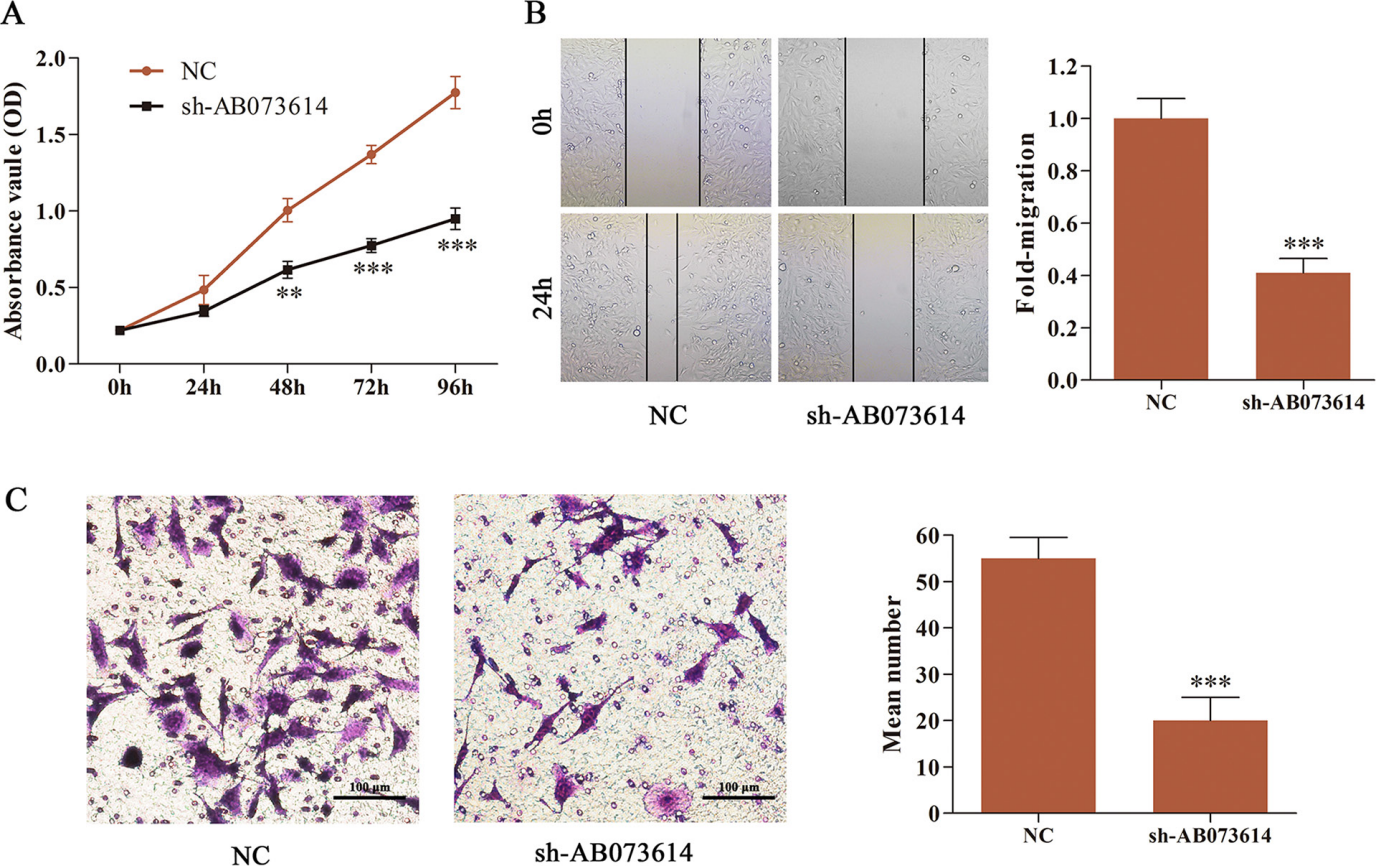
Silencing AB073614 suppressed cell proliferation, migration, and invasion of glioma cells (**A**) Effect of AB073614 knockdown on U251 cell proliferation as measured by MTT assay. Absorbance was read at 490 nm with averages from triplicate wells. (**B**) Knockdown of AB073614 dramatically reduced U251 cell migration as measured by wound healing assay. (**C**) Invaded cells significantly decreased after sh-AB073614 transduction as measured by transwell assay (**P < 0.01; ***P < 0.001).

AB073614 expression was knocked down in U251 cells by transducing sh-AB073614 to examine the role of AB073614 in glioma progression. The MTT assay indicated that the decreased expression of AB073614 dramatically inhibited cell proliferation compared with the NC group (Figure 3A). Then, wound healing assay was performed to assess the effect of AB073614 on the migration of U251 cells, demonstrating a significant repression in migration after silencing AB073614 (Figure 3B). Furthermore, the transwell assay showed that numbers of invaded cells were obviously attenuated in the sh-AB073614 group compared with the NC group (Figure 3C).

### SOX7 was frequently downregulated and negatively regulated by AB073614 in glioma

**Figure 4:**
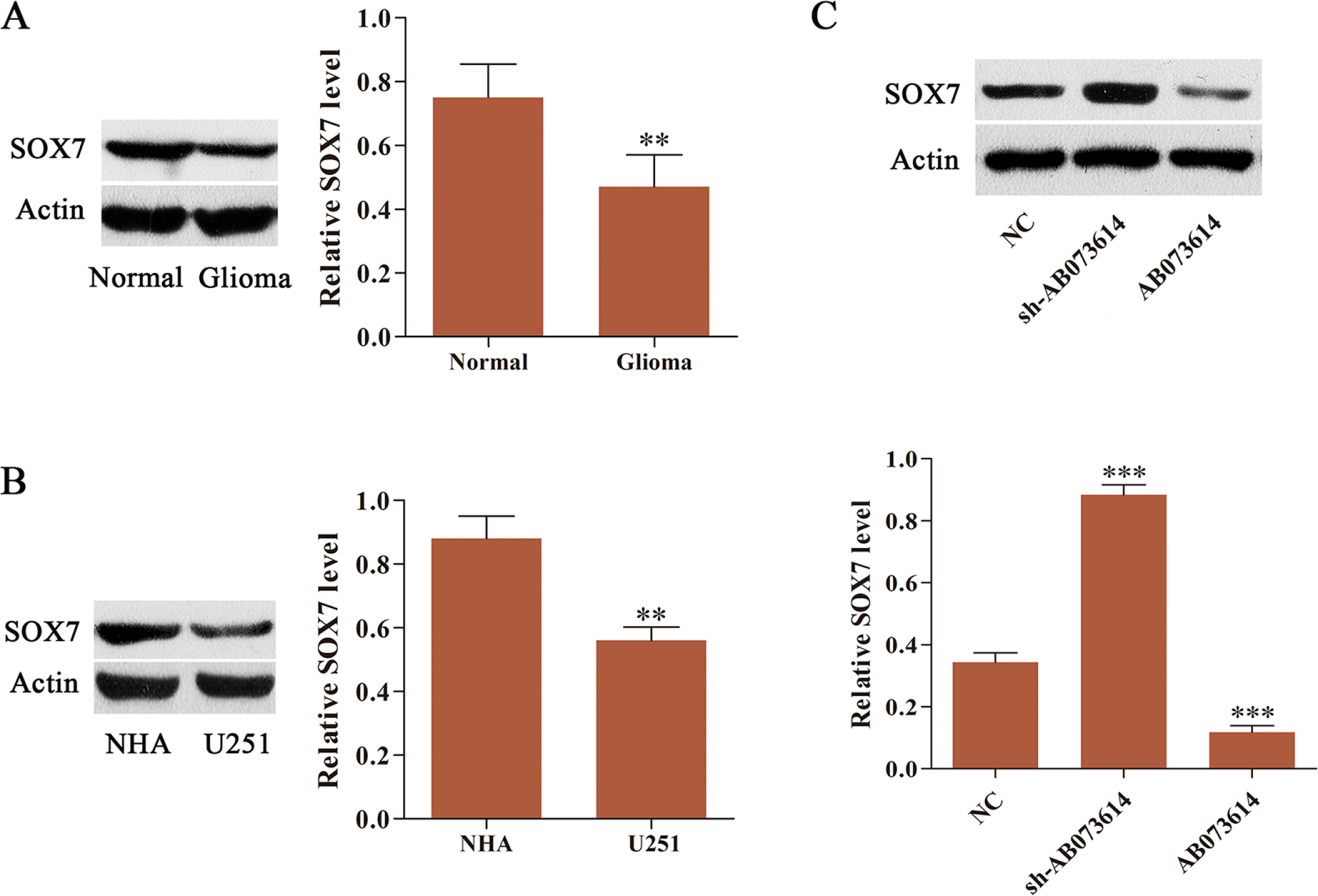
SOX7 expression was frequently downregulated and negatively regulated by AB073614 in glioma (**A**) Western blot analysis of SOX7 expression level in glioma and adjacent normal tissues (*n* = 89). (**B**) Western blot analysis of SOX7 expression level in U251 cells and NHA. (**C**) Western blot analysis showed a remarkably increased expression of SOX7 in U251 cells transduced with sh-AB073614, but a significantly decreased expression of SOX7 in U251 cells transduced with AB073614 compared with the NC group (**P < 0.01; ***P < 0.001).

Emerging studies have suggested that SOX7 is downregulated in several human cancers [19–21]. Therefore, the expression level of SOX7 was examined in both glioma tissues and cells. Western blot showed that SOX7 expression in glioma tissues was lower than that in adjacent normal tissues (Figure 4A). The expression level of SOX7 in U251 cells followed the same trend, being much lower than that in NHA (Figure 4B). NC, sh-AB073614, or AB073614 lentiviral vector was transduced into U251 cells to further investigate the association of AB073614 with SOX7 expression in glioma. The results from western blot analysis revealed that the expression level of SOX7 was greatly downregulated in the AB073614 group but significantly upregulated in the sh-AB073614 group compared with the NC group. These findings confirmed that SOX7 was negatively regulated by AB073614 in glioma (Figure 4C).

### AB073614 regulated Wnt/β-catenin signaling activity dependent on SOX7

**Figure 5:**
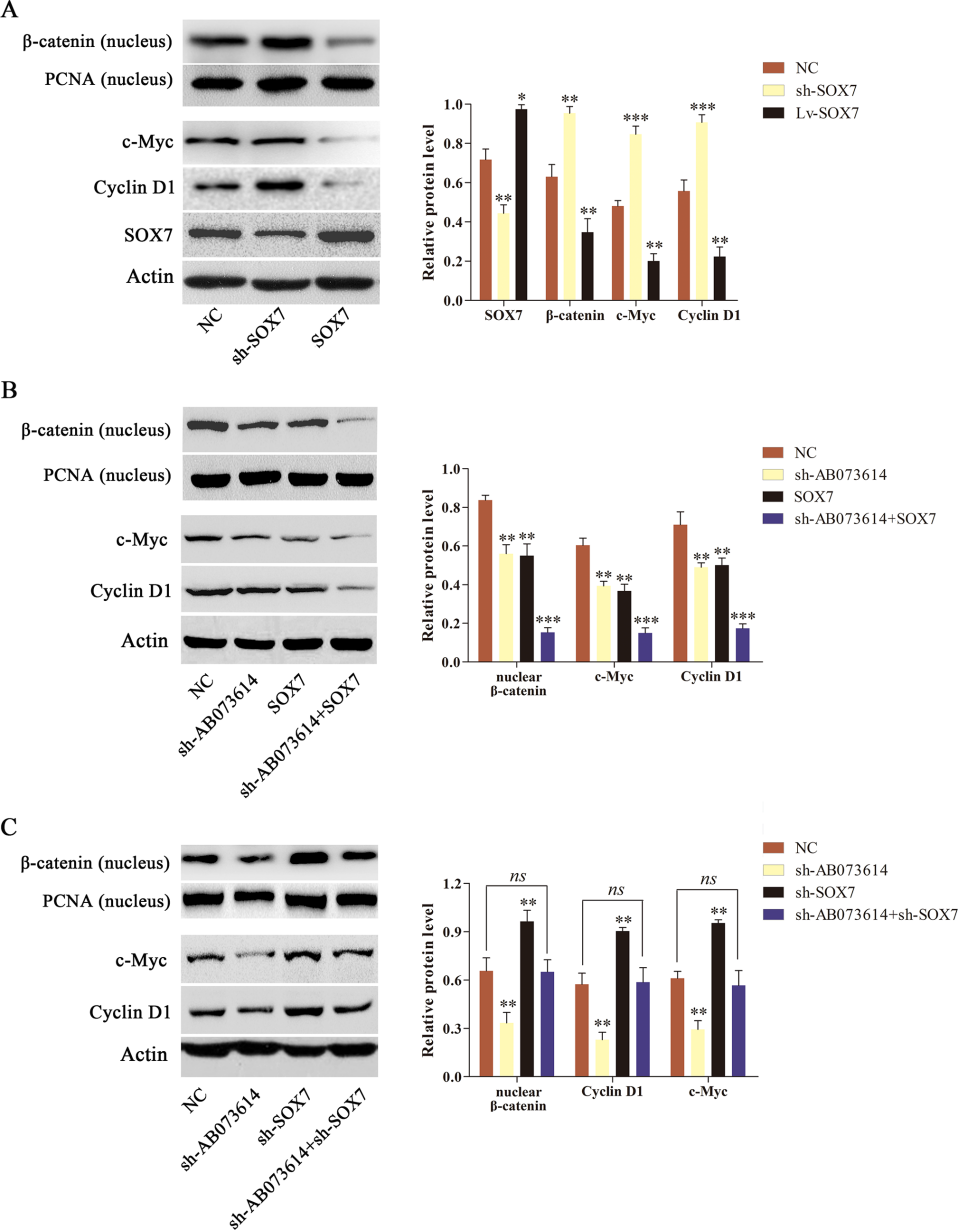
AB073614 regulated the Wnt/β-catenin signaling activity dependent on SOX7 *in vitro* (**A**) The relationship between SOX7 and Wnt/β-catenin signaling activity was investigated using western blot analysis. (**B**) Western blot analysis of the level of nuclear β-catenin and its target gene (Cyclin D1 and c-Myc) expression in U251 cells transduced with NC, sh-AB073614, SOX7, or sh-AB073614 + SOX7 lentiviral vector. (**C**) Western blot analysis of the level of nuclear β-catenin and its target gene (Cyclin D1, c-Myc) expression in U251 cells transduced with NC, sh-AB073614, sh-SOX7, or sh-AB073614 + sh-SOX7 lentiviral vector (*P < 0.05; **P < 0.01; ***P < 0.001).

AB073614 positively regulated the activity of Wnt/β-catenin signaling pathway and negatively regulated the SOX7 expression level in glioma cells. Given that SOX7 has been showed to remarkably downregulate Wnt/β-catenin transcription and inhibit the expression of Wnt target genes, it was hypothesized that AB073614 regulated Wnt/β-catenin signaling activity through SOX7. The relationship between SOX7 and Wnt/β-catenin signaling activity was first confirmed to test this hypothesis. U251 cells were transduced with NC, shRNA targeting SOX7 (sh-SOX7), or SOX7 overexpression (SOX7) lentiviral vector. The transduction efficiency was shown in Figure 5A. Besides, the inhibition of SOX7 with sh-SOX7 induced a significant increase in the level of nuclear β-catenin and its target gene (Cyclin D1 and c-Myc) expression, while SOX7 overexpression contributed to a significant decrease in the level of the three proteins compared with the NC group (Figure 5A). The findings showed a trend of inverse expression pattern between SOX7 and Wnt/β-catenin signaling pathway in glioma. U251 cells were transduced with NC, sh-AB073614, SOX7, or sh-AB073614 + SOX7 lentiviral vector to further validate the hypothesis. Western blot analysis showed a dramatic decrease in the level of nuclear β-catenin and its target gene (Cyclin D1 and c-Myc) expression in both the sh-AB073614 and SOX7 groups compared with the NC group, and a greater decrease in the sh-AB073614 + SOX7 group than in the sh-AB073614 group or SOX7 group (Figure 5B). Moreover, U251 cells were transduced with NC, sh-AB073614, sh-SOX7, or sh-AB073614 + sh-SOX7 lentiviral vector. The results showed a significant decrease in nuclear β-catenin and its target gene (Cyclin D1, c-Myc) expression in the sh-AB073614 group but a significant increase in the sh-SOX7 group compared with the NC group. However, the change trend diminished in the sh-AB073614+sh-SOX7 group. (Figure 5C). Taken together, these results indicated that AB073614 regulated Wnt/β-catenin signaling activity by downregulating SOX7 expression in glioma.

### Inhibition of SOX7 could reverse the tumor-suppressive effect of silencing AB073614 on glioma *in vitro* and *in vivo*

**Figure 6:**
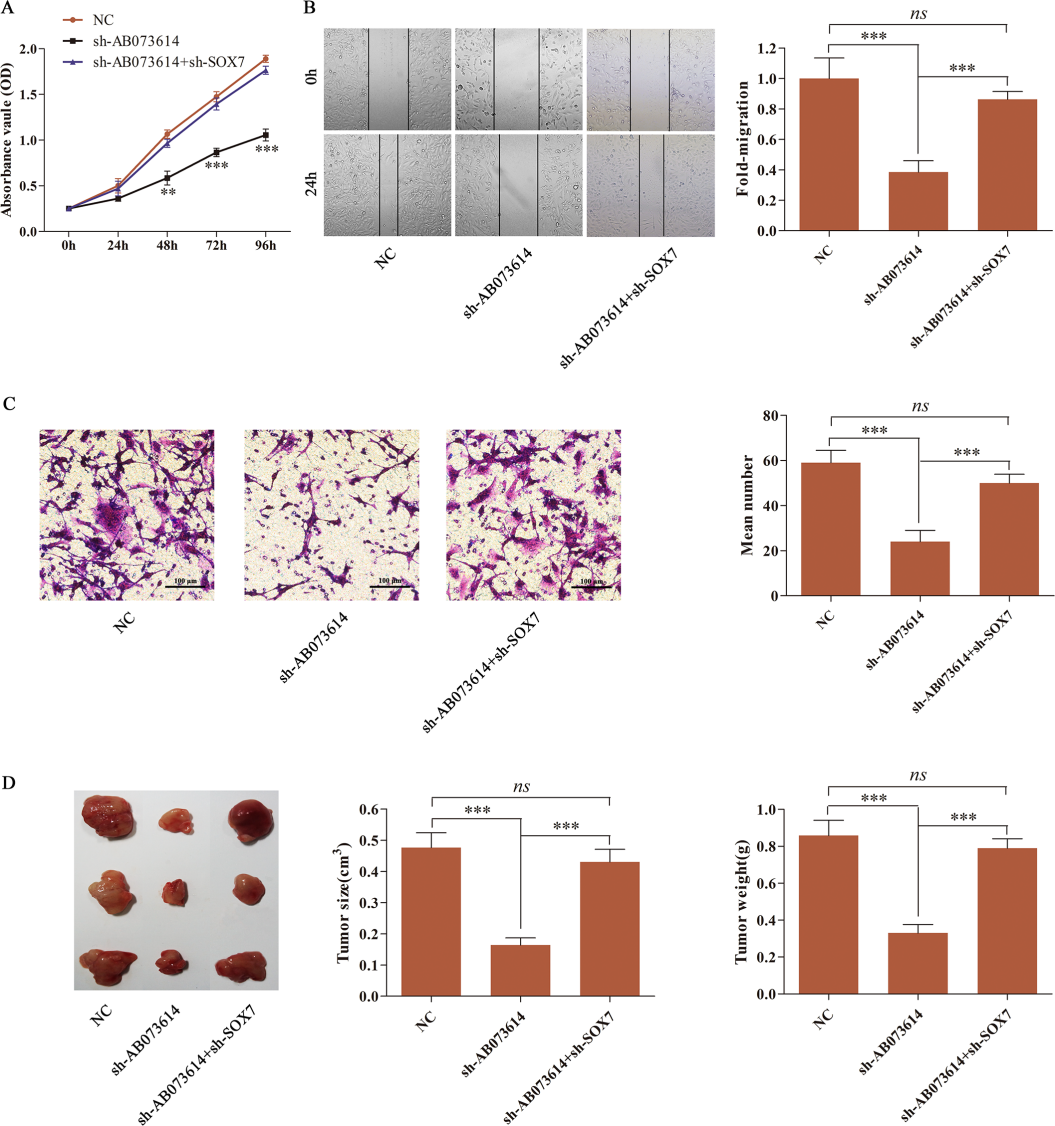
Inhibition of SOX7 abolished the sh-AB073614 induced tumor-suppressive effect on glioma *in vitro* and *in vivo* (**A**–**C**) MTT assay, wound healing assay, and transwell assay were respectively performed to detect the proliferation, migration, and invasion of U251 cells after being transduced with NC, sh-AB073614, or sh-AB073614 + sh-SOX7 lentiviral vector. (**D**) Photographs of isolated tumor tissues 28 days after being inoculated subcutaneously into nude mice with U251 cells transduced with NC, sh-AB073614, or sh-AB073614 + sh-SOX7 lentiviral vector. The tumor volume and weights were measured at 28 days after inoculation (**P < 0.01; ***P < 0.001).

U251 cells were first transduced with NC, sh-AB073614 or sh-AB073614+sh-SOX7 lentiviral vector to further validate the novel pathway in glioma tissues and cells. MTT assay, wound healing assay, and transwell assay were conducted to assess U251 cell proliferation, migration, and invasion. The results showed that silencing AB073614 significantly suppressed cell proliferation, migration, and invasion compared with the NC group. However, the inhibition of SOX7 reversed the tumor-suppressive effect of silencing AB073614 in the sh-AB073614 + sh-SOX7 group (Figure 6A, 6B, and 6C). Then, tumor formation assays were performed in nude mice to explore the pathway *in vivo*. U251 cells transduced with NC, sh-AB073614, or sh-AB073614 + sh-SOX7 lentiviral vector were inoculated subcutaneously into the flanks of nude mice. Tumor formation was then monitored for 28 days. It was found that the average size and weight of tumor in the sh-AB073614 group were much smaller than those in the NC group, indicating a significant tumor-suppressive effect of silencing AB073614 *in vivo*. In contrast, the tumor size and weight in the sh-AB073614 + sh-SOX7 group showed no difference compared with the NC group (Figure 6D). Overall, the tumor inhibition effect of sh-AB073614 was abolished when SOX7 was deleted.

## DISCUSSION

Glioma, especially GBM, is associated with significant mortality due to either metastasis or recurrent disease. Despite great improvement in therapeutic approaches, clinical outcomes of patients with glioma remain poor [22, 23]. The improvement in the survival rate requires a further understanding of pivotal pathogenic mechanisms underlying the initiation and development of glioma. It is thus a huge challenge of current basic and clinical research to seek novel molecular markers for more accurate and efficient treatment of glioma. The present study aimed to investigate the function of AB073614 in glioma. Also, attempts were made to explore the underlying mechanisms of AB073614 and find out a potential therapeutic target for glioma. The findings might contribute to supporting the important role of lncRNAs in the field of tumor research.

At present, an increasing number of studies have focused on the role of lncRNAs in the development of human diseases, especially cancer, such as esophageal squamous cell carcinoma [24], non-small cell lung cancer [25], breast cancer [26], gastric cancer [27], and colorectal cancer [28], etc. However, for most lncRNAs, including AB073614, the potential molecular mechanisms of biological function and signaling pathways by which lncRNAs exert their biological activity remain largely unknown. It has been reported that AB073614 may act as a functional oncogene in ovarian cancer development by targeting ERK1/2- and Akt-mediated signaling pathway [9]. Moreover, a previous clinical study demonstrated that AB073614 expression was significantly upregulated in glioma tissues compared with normal brain tissues, and the upregulation of AB073614 could be an independent predictor of poor prognosis in patients with glioma [10]. Of note, a recent study found that AB073614 was significantly upregulated in glioma tissues and cell lines, and the knockdown of AB073614 inhibited epithelial–mesenchymal transition phenotype in glioma cells [29], whereas the underlying mechanisms were not explored. Consistent with these observations, the expression of AB073614 in glioma was first examined in the present study, and AB073614 was found to be frequently upregulated in glioma tissues and cell lines compared with adjacent normal brain tissues and NHA, respectively. More importantly, the level of AB073614 expression was positively correlated with the clinical WHO grade. Therefore, it was speculated that AB073614 might be a critical regulator in the development of glioma. As expected, silencing AB073614 was found to significantly suppress cell proliferation, migration, and invasion of glioma cells. These results revealed that AB073614 might function as a potent tumor promoter in glioma. However, the underlying molecular mechanisms remained unclear. Therefore, the molecular mechanisms by which AB073614 exerted its functions *in vitro* and *in vivo* were investigated.

Many studies have demonstrated that SOX7, as a tumor suppressor, is involved in a variety of human cancers, including gliomas [30, 31]. However, whether and how SOX7 is regulated by lncRNAs in glioma is still unknown. Interestingly, upregulated AB073614 was concomitant with underexpressed SOX7 in both glioma tissues and cell lines. After U251 cells were transduced with AB073614 or sh-AB073614 lentiviral vectors, SOX7 expression was significantly decreased or increased, respectively. These findings confirmed that SOX7 expression was negatively regulated by AB073614 in glioma cell lines. Moreover, the inhibition of SOX7 expression significantly reversed the tumor-suppressive effects of silencing AB073614 on glioma cell proliferation, migration, and invasion *in vitro*. Tumor formation assay was performed to further validate the AB073614/SOX7 signaling pathway *in vivo*. The results showed a consistent trend that the inhibitory effect of sh-AB073614 on tumor formation was abolished when SOX7 was deleted, indicating that the tumor-suppressive effect of silencing AB073614 was dependent on the presence of SOX7 in glioma.

Mounting evidences have documented that the aberrant activation of Wnt/β-catenin signaling pathway was implicated in the development of several human malignancies [32, 33]. β-Catenin is the central signal transduction molecule in the Wnt signaling pathway. It is a critical step of β-catenin nuclear translocation, and accumulation of nuclear β-catenin is a vital event in the activation of the Wnt pathway. β-Catenin interacts with downstream transcription factors in the nucleus to form β-catenin–TCF/LEF transcriptional complexes, in turn regulating the expression of its gene products including Axin2, LEF-1, c-Myc, Cyclin D1, and so on [34]. Numerous studies have confirmed that nuclear β-catenin plays a dominant role in tumor progression through regulating cell cycle transition and cell apoptosis [35]. The cytoplasm and nucleus were isolated in the present study and nuclear β-catenin expression was determined using western blot assay. In agreement with previous studies, the results showed that the expression levels of nuclear β-catenin, as well as its downstream targets Cyclin D1 and c-Myc, were markedly increased in glioma tissues and cell lines. The findings confirmed that the Wnt/β-catenin signaling pathway was activated in human glioma tissues and cells.

A previous study showed that SOX7 might be a negative regulator of Wnt/β-catenin signaling pathway through disrupting the transcriptional activity of β-catenin–TCF/LEF complex and its downstream targets, such as Cyclin D1, c-Myc, and FGF9 in endometrial cancer [18]. Consistently, Cui et al. [36] demonstrated that SOX7 played a crucial role in inhibiting tumorigenesis and progression by repressing the Wnt/β-catenin signaling pathway in gastric cancer. Liu recently et al. [37] found that SOX7, together with AXIN2, as a potential co-regulator of the Wnt/β-catenin signaling pathway by targeting Smad7, played an important role in controlling breast cancer progression. Of great interest, SOX7 was reported to downregulate Wnt/β-catenin transcription and decrease the expression of Cyclin D1 and c-Myc in glioma [38]. These findings suggested that SOX7 might serve as a tumor suppressor through suppressing the Wnt/β-catenin signaling pathway in multiple tumors, including glioma. Consistent with previous findings, the results of the present study indicated that SOX7 deficiency significantly increased the level of nuclear β-catenin and its target gene (Cyclin D1 and c-Myc) expression, while SOX7 overexpression significantly decreased the level of these three proteins, showing a trend of inverse expression pattern between SOX7 and Wnt/β-catenin as well as its target genes in glioma. Further, a significant decrease in the level of nuclear β-catenin and its target gene (Cyclin D1 and c-Myc) expression in U251 cells transduced with sh-AB073614. However, such a decrease was significantly rescued by sh-SOX7. Moreover, the levels of nuclear β-catenin and its target gene (Cyclin D1 and c-Myc) expression were much lower in U251 cells transduced with sh-AB073614 + SOX7 than in U251 cells transduced with sh-AB073614 or SOX7 only. Taken together, these results strongly suggested that the tumor-suppressive effect of silencing AB073614 was, in large part, owing to its ability to activate SOX7 and subsequently inhibit the activation of Wnt/β-catenin signaling pathway.

In conclusion, the findings demonstrated that AB073614 expression was upregulated in both glioma tissues and cell lines. Furthermore, silencing AB073614 could significantly inhibit glioma cell proliferation, migration, and invasion. Finally, the results suggested that AB073614 was an oncogenic lncRNA that promoted the progression of glioma by activating the Wnt/β-catenin signaling pathway via suppression of SOX7. The findings might provide new insights into the molecular mechanisms of the formation and progression of human glioma. It is believed that silencing AB073614 might be a potential therapeutic strategy for glioma treatment in the near future.

## MATERIALS AND METHODS

### Patients and specimens

A total of 89 paired glioma tissue samples and the corresponding adjacent normal brain tissues were collected during the period 2013–2015 in Tangdu Hospital, Fourth Military Medical University, Xi’ an, China. Tissue samples were immediately frozen in liquid nitrogen and stored at –80°C for extracting RNA and proteins. None of the patients had ever suffered from neurosurgery, chemotherapy, or radiotherapy. According to the WHO classification, all patients were divided into four histopathological grades: grade I, II, III, and IV. The study was approved by the Ethics Committee of Tangdu Hospital, and written informed consent was obtained from each patient.

### Cell culture and transduction

NHA and the human glioma cell line (U251) were acquired from Beijing Zhongyuan Company (Beijing, China) and grown in Dulbecco’s modified Eagle’s medium (DMEM; Hyclone, UT, USA) supplemented with 10% heat-inactivated fetal bovine serum (FBS; Gibco, CA, USA), streptomycin (100 µg/mL), and penicillin (100 units/mL). All cell lines were maintained at 37°C in a humidified atmosphere containing 5% CO_2_. U251 cells were cultured in six-well plates and transfected with shRNA targeting AB073614, pcDNA-AB073614, shRNA targeting SOX7, pcDNA-SOX7, PAX2, PMD2G, and pcDNA lentiviral vectors as NC in DMEM without any FBS. After incubation for 6 h, 2 mL of medium with 10% FBS was added to the cells to replace transfection solution. After 48 h, the lentiviral supernatants were filtered and added to the U251 cells.

### Western blotting

Proteins from glioma tissue samples and cell line (U251) were separated by sodium dodecyl sulfate polyacrylamide gel electrophoresis and transferred on to polyvinylidene membranes. The blots were incubated with Cyclin D1 primary antibody (Cell Signaling, MA USA), c-Myc primary antibody (Cell Signaling), and β-catenin (Abcam, Cambridge, UK). Then, the blots were incubated with an appropriate second antibody that conjugated with the horseradish peroxidase for 2 h. Finally, the complexes were detected using chemiluminescence.

### Quantitative real-time PCR

Target genes were analyzed using an Mx4000 or Mx3005 multiplex qRT-PCR system. Then, the expression of AB073614 was normalized to glyceraldehyde-3-phosphate dehydrogenase (GAPDH) mRNA level. The primary sequences used were as follows: AB073614: forward, 5ʹ-TCTGCTCCTGGGTCTTACAC-3ʹ; AB073614: reverse, 5ʹ-TGCAACCACATGTAACCACA-3ʹ; GAPDH: forward, 5ʹ-CCCATCACCATCT¬TCCAGGAG-3ʹ; GAPDH: reverse, 5’-GTTGTCATGGATGAC¬CTTGGC-3ʹ.

### MTT assay

U251 cells were seeded in 96-well plates. The cell viability was analyzed using MTT assay. After incubation, the cells were treated with MTT (5 mg/mL) for 4 h at 37°C. Then, the medium was removed and dimethyl sulfoxide was added to each well. The optical density values of the cells were determined on a microplate reader at 490 nm.

### Transwell assay of cell invasion

Yang et al reported the methods of transwell assay [21]. In brief, Transwell insert chamber coated with Matrigel (BD Biosciences, NJ, USA) was used according to the manufacturer’s detailed instruction. The invading cells were fixed on the bottom side with 4% polyoxymethylene after 24 h of incubation. At last, the invading cells on the bottom side were stained with 0.1% crystal violet. The cell counting was conducted under a microscope at 100× magnification.

### Wound healing assays

The cells were cultured in six-well plates and then incubated until 80% confluence before of wounding. Then, a 200-μL tip was used to make a vertical wound and the cells were washed three times with phosphate-buffered saline to remove the cell debris. Cell migration to the wounded region was observed by Nikon microscopy at the designated time point.

### Statistical analysis

All the statistical analyses were conducted using Statistical Package for the Social Sciences Version 20.0 software (SPSS Inc., IL, USA). One-way analysis of variance (ANOVA) followed by the Bonferroni test was used to analyze the data. The values were expressed as the mean ± standard error of mean. Survival analysis was carried out using the log-rank test in GraphPad Prism 5. *P* values < 0.05 were considered statistically different (*P < 0.05; **P < 0.01; ***P < 0.001).
